# Oxytocin Protects Hippocampal Memory and Plasticity from Uncontrollable Stress

**DOI:** 10.1038/srep18540

**Published:** 2015-12-21

**Authors:** Sun-Young Lee, Seong-Hae Park, ChiHye Chung, Jeansok J. Kim, Se-Young Choi, Jung-Soo Han

**Affiliations:** 1 Department of Biological Sciences, Konkuk University, 120 Neungdong-ro, Gwangjin-gu, Seoul 05029, Republic of Korea; 2 Department of Physiology and Dental Research Institute, Seoul National University School of Dentistry, Seoul 03080, Republic of Korea; 3 Department of Psychology, University of Washington, Seattle, WA 98195-1525, USA

## Abstract

The hippocampus is vulnerable to uncontrollable stress and is enriched with oxytocin receptors, but their interactive influences on hippocampal functioning are unknown. This study aimed to determine the effects of intranasal oxytocin administration on stress-induced alterations in synaptic plasticity and spatial memory in male rats. While vehicle-administered stressed rats showed impairment in long-term potentiation, enhancement in long-term depression, and weakened spatial memory, these changes were not observed in oxytocin-administered stressed rats. To reveal the potential signaling mechanism mediating these effects, levels of phosphorylated extracellular signal-regulated kinases (pERK) in the hippocampus was examined. Western blotting showed that oxytocin treatment blocked stress-induced alterations of pERK. Additionally, the oxytocin receptor antagonist L-368,899 inhibited the oxytocin’s protective effects on hippocampal memory to stress. Thus, intranasal administration of oxytocin reduced stress effects on hippocampal synaptic plasticity and memory in rats via acting on oxytocin receptors and regulating ERK activity. This study suggests that exogenous oxytocin may be a therapeutically effective means to counter the detrimental neurocognitive effects of stress.

It is well documented that stress, a biologically significant and pervasive environmental factor, can have lingering negative effects on memory functions in the hippocampus^1^,^2^. It has been postulated that during *uncontrollable* stress experiences, animals and humans learn that their actions have no bearing over the aversive outcome, and that such learning, known as ‘learned helplessness’, results in neurophysiological changes that modify subsequent behavior^3^.

Consistent with this notion, rats that experienced unpredictable and inescapable shocks while being immobilized perform poorly on hippocampal memory tasks, and their hippocampi display alterations in long-term potentiation (LTP) and long-term depression (LTD)^4^,^5^,^6^,^7^, two putative synaptic models of learning and memory^8^. Because various psychopathologies, such as anxiety disorders, depression, and drug use relapse^9^, incorporate stress-related cognitive disturbances, identifying the signaling pathway in the hippocampus affected by stress has a wide-ranging clinical significance.

Recently, the neuropeptide oxytocin has been implicated in modulating complex social (e.g., empathy, trust) and emotional (e.g., anxiety) behaviors in humans and animals^10^,^11^,^12^. In rats (both sexes), acute oxytocin administration, via nasal and intraperitoneal routes, has been reported to promote prosocial behaviors^11^ while diminishing the impacts of stressors as measured by corticosterone^13^,^14^,^15^, a rodent analog of human cortisol elevated during stress. However, the neurophysiological basis of oxytocin’s anti-stress effects on behavior remains relatively unknown. Thus, we investigated whether oxytocin, administered intranasally^16^,^17^, modifies the uncontrollable stress effects on the hippocampal plasticity and memory. Because MEK-extracellular signal-regulated kinase (ERK) signaling in hippocampus is involved in regulation of both stress and the action of oxytocin^18^,^19^, we further examined whether oxytocin counters stress effects on the MEK-ERK signaling pathway in the hippocampus.

## Results

### Effects of intranasal oxytocin administration on stress-induced impairments in hippocampal synaptic plasticity

**Figure 1:**
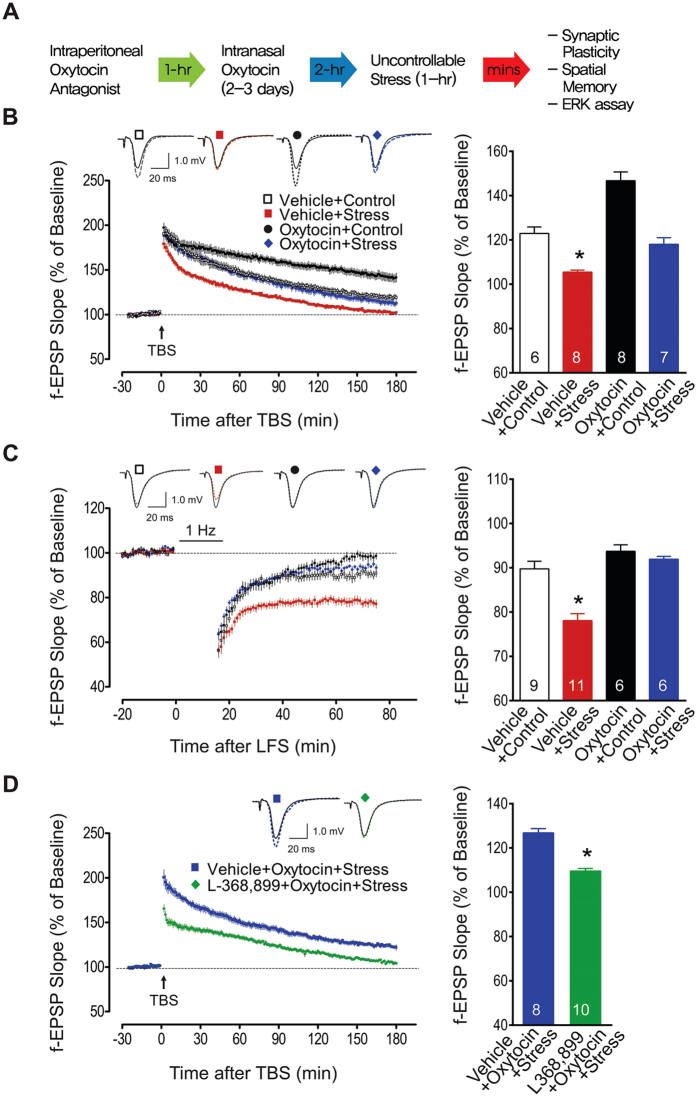
Oxytocin, stress, and hippocampal synaptic plasticity. (**A**) General experimental procedure (detailed methods). (**B**) Effects of oxytocin and stress on subsequent CA1 LTP *in vitro*. TBS of 10 stimulus trains (4 pulses at 100 Hz) delivered at 5 Hz was used to induce long-lasting LTP. Hippocampal slices from vehicle + stress animals exhibited markedly impaired LTP than those from other groups (*p < 0.05). Top traces show the representative average of 10 consecutive f-EPSPs before (solid lines) and after (dotted lines) TBS from four groups. Scale bar represents 20 ms and 1.0 mV. The bar graph shows the mean (±SEM) normalized slope 120–180 m after TBS: vehicle + control (122.9 ± 2.9%), vehicle + stress (105.5 ± 0.9%), oxytocin + control (146.7 ± 4.0%), and oxytocin + stress (118.0 ± 3.3%) groups. (**C**) Hippocampal slices from vehicle + stress animals showed greater CA1 LTD following LFS (900 pulses, 1 Hz) than those from other groups (*p < 0.05). The top traces show the representative average of 10 consecutive f-EPSPs before (solid) and after LFS (dotted line). The bar graph shows the mean (±SEM) normalized slope 40–60 m after LFS from vehicle + control (89.8 ± 1.7%), vehicle + stress (78.1 ± 1.6%), oxytocin + control (93.7 ± 1.5%), and oxytocin + stress (91.9 ± 0.6%) groups. (**D**) Reversal of oxytocin’s effects by prior administration of the oxytocin receptor antagonist L-368,899 (109.5 ± 1.2%) compared to vehicle (126.8 ± 1.9%; *p < 0.05). The number in the bar graph indicates the number of brain slices per group. Abbreviations: f-EPSP, field excitatory postsynaptic potential; LFS, low frequency stimulation; LTD, long-term depression; LTP, long-term potentiation; TBS, theta burst stimulation.

Hippocampal LTP and LTD were assessed in rats assigned to one of four groups (a 2 × 2 factorial design; intranasal drug x stress): vehicle + control, vehicle + stress, oxytocin + control, and oxytocin + stress (Fig. 1A). As previously reported^6^, 60-min of restraint plus variable tailshocks impaired Schaffer collateral/commissural-CA1 LTP *in vitro* in vehicle + stress animals. Specifically, following TBS, the f-EPSP slopes recorded from vehicle + stress slices were significantly impaired compared to those from vehicle + control, oxytocin + control and oxytocin + stress groups (two-way ANOVA; main effect of stress: *F*_1,25_ = 61.50, *P* < 0.0001; main effect of drug: *F*_1,25_ > 26.21, *P* < 0.0001; interaction effect, *F*_1,25_ = 3.62, *P* = 0.07). *Post-hoc* analyses revealed that LTP impairments were not observed in oxytocin + stress slices (*P* < 0.05, compared to vehicle + stress slices; Fig. 1B).

In contrast to LTP, hippocampal slices from the same vehicle + stress group showed enhanced LTD following low frequency stimulation^20^ of the Schaffer collateral/commissural-CA1 pathway (two-way ANOVA; main effect of stress: *F*_1,28_ = 17.02, *P* < 0.0001; main effect of oxytocin: *F*_1,28_ = 29.84, *P* < 0.0001; interaction effect: *F*_1,28_ = 9.17, *P* = 0.005; Fig. 1C). This effect on LTD was prevented in animals given intranasal oxytocin prior to stress (*P* < 0.05). These findings of oxytocin pretreatment preventing stress effects on LTP (i.e., impairment) and LTD (i.e., enhancement) indicate anti-stress properties of oxytocin on hippocampal plasticity.

Corticosterone immunoassay of blood collected after 1 h acute stress revealed significantly higher corticosterone levels (*F*_1,9_ = 112.87, *P* < 0.0001) irrespective of the drug pretreatment (*F*_1,9_ = 1.89, *P* = 0.20; Table 1). This indicates that intranasal oxytocin does not impede stress-induced corticosterone secretion.

We then examined whether intraperitoneal injection of the oxytocin receptor antagonist L-368,899 (5 mg/kg) can prevent intranasal oxytocin’s protective effects on hippocampal plasticity (Fig. 1D). L-368,899 has been found to cross the blood-brain barrier (BBB) and be measureable in the CSF for several hours^21^. Hippocampal slices from oxytocin + stress animals pretreated with L-368,899 exhibited impaired LTP compared to those from oxytocin + stress animals pretreated with vehicle (unpaired t-test; *t*_16_ = 7.94, two-tailed *P* < 0.0001). Hence, intranasal oxytocin utilizes its cognate receptors for mediating anti-stress effects on hippocampal plasticity.

### Effects of intranasal oxytocin administration on stress-induced impairments in hippocampal memory

**Figure 2:**
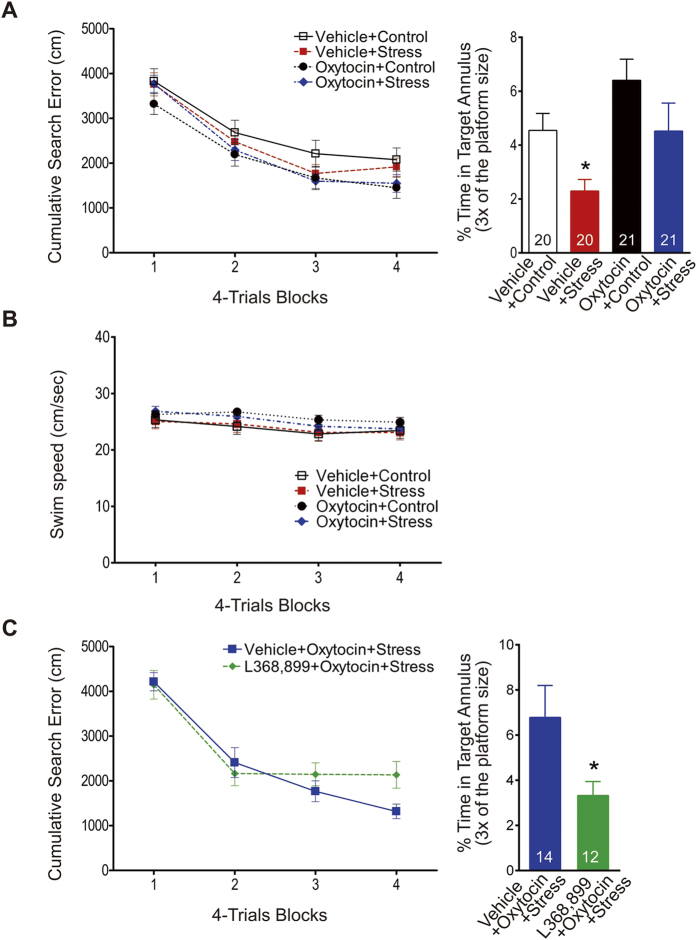
Oxytocin, stress, and spatial memory. (**A**) *Left*, cumulative search error in finding a submerged platform over four blocks of four trials/block. *Right*, percentage of time spent in the annulus target (3X the platform diameter size) when the platform was removed and rats probed the next day. The stress-induced impairment of spatial memory retention was prevented by intranasal oxytocin treatment (*p < 0.05). (**B**) Swimming speed during training trials. (**C**) Prior administration of the oxytocin receptor antagonist L-368,899 blocked oxytocin’s anti-stress effects on spatial memory (*p < 0.05).

To assess behavioral effects, separate groups of animals were tested in a Morris water maze task. All animals improved in locating the submerged platform across the 16 training trials, as shown by decreasing search errors (Fig. 2A; three-way ANOVA with 4 trials/block as a repeated measure; main effect of training: *F*_3,234_ = 103.10, *P* < 0.0001). Overall, animals pretreated with oxytocin performed reliably better than those pretreated with vehicle (main effect of drug: *F*_1,78_ = 3.89, *P* = 0.05). Neither the main effect of stress nor the interaction effects of drug x stress, drug x training, stress x training, and drug x stress x training were significant. The probe test next day, assessing the percentage of time within the annulus target, showed that spatial memory retention was impaired by stress and improved by oxytocin (two-way ANOVA; main effect of stress: *F*_1,78_ = 8.73, *P* = 0.004; main effect of drug: *F*_1,78_ = 6.78, *P* = 0.011; interaction effect: *F*_1,78_ = 0.22, *P* = 0.64; Fig. 2A). *Post-hoc* analyses revealed that the oxytocin + stress group showed significantly better retention of spatial memory for the trained platform location than the vehicle + stress group (*P* < 0.05). In addition, effects of the stress and oxytocin on the locomotor activity were examined with swimming speed during training trials. Neither the main effects of stress and drug nor the interaction effects of drug x stress, drug x training, stress x training and drug x stress x training were significant (Fig. 2B). Only the main effect of training was significant *F*_3,234_ = 20.02, *P* < 0.0001.

When animals were administered L-368,899 prior to intranasal oxytocin, the anti-stress effect of oxytocin on spatial memory was negated (Fig. 2C). Specifically, vehicle + oxytocin + stress and L368,899 + oxytocin + stress groups improved comparably in locating the submerged platform across the 16 training trials (two-way ANOVA with 4-trials/block as a repeated measure; main effect of training: *F*_3,72_ = 45.39, *P* < 0.0001). Neither the main effect of group (*F*_1,24_ < 1.0) nor the group x training interaction (*F*_3,72_ = 2.05, *P* = 0.11) were significant. However, on the probe test, the L-368,899 animals showed significantly weaker retention of spatial memory compared to the vehicle animals (unpaired t-test; *t*_*24*_ = 2.12, two-tailed *P* = 0.045, Fig. 2C).

### Effects of intranasal oxytocin administration on stress-induced ERK alteration in hippocampus

**Figure 3:**
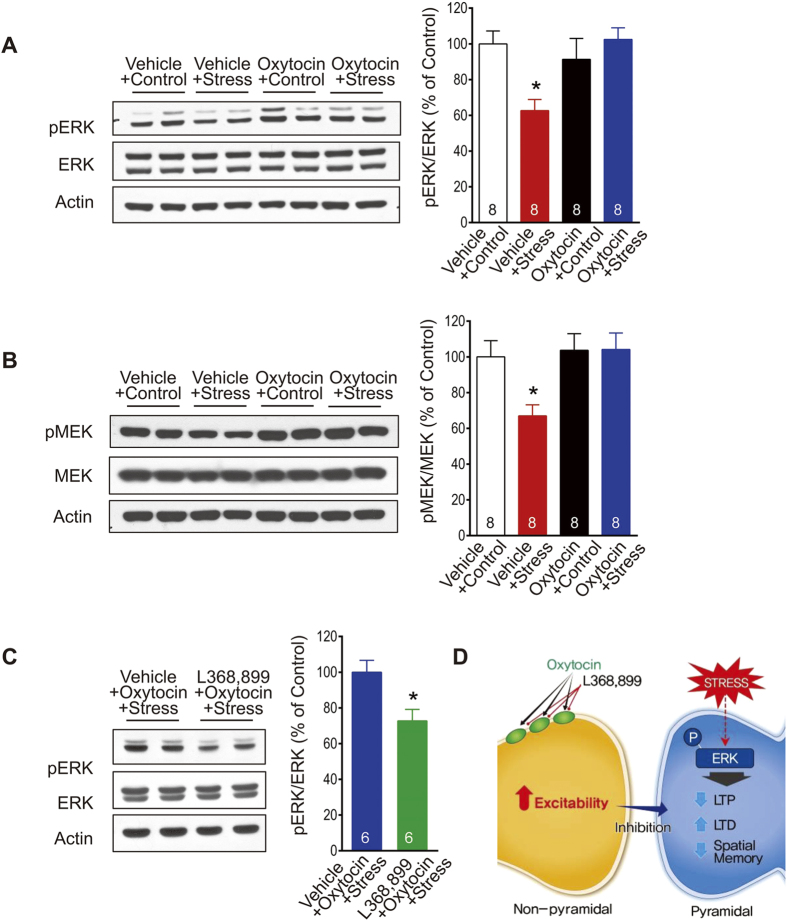
Oxytocin, stress, and extracellular signal-regulated kinase (ERK) signaling. (**A**) *Left*, representative Western blots of hippocampal ERK and phosphorylated ERK (pERK). *Right*, the stress-induced reduction in hippocampal pERK levels was prevented by intranasal oxytocin treatment (*p < 0.05). (**B**) *Left*, representative Western blots of hippocampal MEK and phosphorylated MEK (pERK). *Right*, the stress-induced reduction in hippocampal pMEK levels was prevented by intranasal oxytocin treatment (*p < 0.05). (**C**) The anti-stress effect of oxytocin on hippocampal pERK levels was blocked by prior administration of the oxytocin receptor antagonist L-368,899 (*p < 0.05). The number in the bar graph represents the number of animals per group. (**D**) A hypothetical model describing the effects of oxytocin on hippocampal neurons and the ERK signaling pathway to restore stress-induced deficits in synaptic plasticity and memory.

Because stress alters the ERK pathway^18^, we examined whether oxytocin might dampen the stress effects on the hippocampus through the ERK signaling. To test this, hippocampal pERK and pMEK levels were measured immediately after oxytocin and stress treatments. A previous study showed that pERK levels, measured immediately after termination of uncontrollable stress, were reduced in both cytosol and nucleus^18^. Cytoplasmic levels of pERK revealed no main effects of stress (*F*_1,28_ = 2.54, *P* = 0.12) and drug (*F*_1,28_ = 3.59, *P* = 0.07), but a significant stress x drug interaction (*F*_1,28_ = 8.70, *P* = 0.006). *Post-hoc* analyses indicated that hippocampi of oxytocin + stress rats exhibited higher levels of pERK than those of vehicle + stress rats (*P* < 0.05, Fig. 3A). pMEK signaling (upstream of ERK) showed neither main effect of stress (*F*_1,28_ = 3.67, *P* = 0.07) nor stress x drug interaction (*F*_1,28_ = 3.85, *P* = 0.06), but main effect of drug was significant (*F*_1,28_ = 5.77, *P* = 0.02). More importantly, similar to results of hippocampal pERK levels, *post-hoc* analyses indicated that hippocampi of oxytocin + stress rats exhibited higher levels of pMEK than those of vehicle + stress rats (*P* < 0.05, Fig. 3B). Pretreatment with L-368,899 blocked oxytocin’s anti-stress effect on the pERK level (unpaired t-test; *t*_*10*_ = 2.95, two-tailed *P* = 0.014, Fig. 3C). Thus, oxytocin’s ability to reverse stress effects on hippocampal plasticity and memory might, at least partly, be through the MEK-ERK signaling^19^.

## Discussion

Unpredictable and inescapable stress alters hippocampal plasticity and impairs hippocampal dependent memory^4^,^5^,^6^,^7^. On the other hand, oxytocin has been reported to enhance either the establishment or the maintenance of LTP in the brain areas including hippocampal CA1^19^,^22^,^23^,^24^. Therefore, the present study examined the effects of oxytocin administered intranasally on stress-induced changes in hippocampal synaptic plasticity and hippocampal dependent memory. Consistent with studies mentioned above, we observed that hippocampal plasticity and spatial memory was impaired by stress and that oxytocin administered intranasally enhanced the maintenance of LTP and reduced LTD, at the CA1 region of the hippocampus.

Above all, our results indicate that intranasal administration of oxytocin was effective in preventing stress-induced alterations in hippocampal plasticity and impairments in spatial memory. Given that endogenous oxytocin levels normally increase in response to stress^25^, it is likely that the beneficial effects described herein stem from even greater increases caused by exogenous oxytocin. Moreover, intranasal oxytocin’s anti-stress effects were due to its binding to oxytocin receptors, and not nonspecific effects, because the beneficial effects on hippocampal LTP and spatial memory were effectively blocked by the oxytocin receptor antagonist L-368,899.

Though oxytocin was administered intranasally, its protective effects against stress are likely to be due to changes within the hippocampus. In rats, intranasal delivery has been shown to increase oxytocin in microdialysates in the hippocampus, with the peak level (~200%) occurring ~30–60 min later^16^, and autoradiographic studies have revealed that the hippocampus is one of the brain structures with abundant oxytocin receptors^26^,^27^,^28^,^29^. The fact that pretreatment with the oxytocin receptor antagonist L-368,899, with a half-life of ~2-hr^30^, blocked subsequent oxytocin’s effects is consistent with the view of local actions in the hippocampus.

However, there are a couple of caveats in the study that need to be considered. First, the intranasal oxytocin dosage (200 μl) required to prevent stress effects on hippocampal synaptic plasticity and memory was 10 folds higher than the 20 μl intranasal oxytocin used to detect 200% rise in dialysate oxytocin in the rat hippocampus^16^. The high dosage necessitated in the present study might be due the tilting of the animal’s head during intranasal oxytocin delivery, which can affect the transport dosage from the nasal cavity to the brain^31^, and/or to the severity of the stressor (i.e., 1 hr restraint + 60 intermittent tailshocks) compared to other studies that employed relatively milder stressors (e.g., 10 min noise)^15^. Hence, the possibility of oxytocin preventing stress effects via affecting other brain structures (e.g., the amygdala) and/or via non-centrally cannot be excluded. Similarly, the possibility that systemically administered oxytocin antagonist L-368,899 counteracted intranasal oxytocin effects external to the hippocampus cannot be excluded. Future studies will need to determine whether specific elevation of oxytocin and blockade of oxytocin receptors in the hippocampus can impact stress effects on hippocampal synaptic plasticity and memory.

The question is then how intranasal oxytocin may act to offset the stress effects in the hippocampus? Given that the corticosterone immunoassay of blood collected before and after stress revealed no effect of oxytocin on the stress-induced corticosterone release, this suggests that intranasal oxytocin effects is not due dampening corticosterone and its downstream pathways. Previous studies have shown that central infusion of oxytocin attenuated the stress activations of the hypothalamic-pituitary-adrenal (HPA) axis response (i.e. corticosterone release) and the hippocampal c-fos expression in estrogen-primed ovariectomized female rats^15^,^32^. Anxiety behaviors are also reduced in female rats administered oxytocin intracerebroventricularly^15^. These reports are supported by studies using female oxytocin-deficient mice, which found (i) enhanced anxiety-related behavior in the elevated plus maze task^33^; (ii) enhanced stress-induced corticosterone levels; and (iii) increased c-fos expression in the amygdala following stress^34^,^35^,^36^. Given that stress-induced impairment of hippocampal LTP was prevented by blockade of glucocorticoid receptors^37^,^38^,^39^, it is important that future studies investigate the interactive effects of intranasal oxytocin and stress on the HPA axis activity and hippocampal glucocorticoid signaling.

Extracellular recordings in hippocampal slices have revealed that nonpyramidal neurons, but not the pyramidal neurons, respond to oxytocin^29^,^40^. Specifically, bath applied oxytocin greatly excited nonpyramidal cells while the excitability of pyramidal cells remained virtually unchanged. Other studies have shown that acute stress decreases ERK signaling^18^ which is accompanied by impairment in LTP. If so, the present findings of intranasal oxytocin preventing stress effects in the hippocampus may have occurred via the elevated oxytocin level stimulating the nonpyramidal neurons that increased their inhibition of the pyramidal neurons and thereby preventing the stress-associated decrease in the ERK signaling, which normally would alter synaptic plasticity and impair memory functions in the hippocampus (Fig. 3D). Intranasal oxytocin administration then may offer a potential and practical route of preventive treatment against stress effects on cognitive functions that can lead to various psychopathologies.

## Methods

### Animals

Naïve male Sprague-Dawley rats were singly housed and maintained on a 12-hr light-dark cycle (lights on at 07:00 h) in a climate-controlled vivarium. Animals were acclimatized to the vivarium and handling for 1–2 weeks before the experiments. All experiments were conducted during the light phase of the cycle. And all experimental protocols were approved by the Seoul National University and the Konkuk University Institutional Animal Care and Use Committee. The methods were carried out in accordance with the approved guidelines.

### Stress paradigm

Approximately 2-hrs after the last intranasal administration of oxytocin or vehicle, half of the animals from each drug condition were restrained in a Plexiglas tube and exposed to 60 tail-shocks (1 mA intensity; 1 s duration; 30 to 90 s inter-shock interval). Unstressed control animals were left undisturbed in the cage. The restraint-tail shock stress procedure, adapted from the “learned helplessness” paradigm in which animals are exposed to unpredictable and uncontrollable aversive stimulus^41^, has previously been shown to reliably impair LTP in the hippocampus^4^,^5^,^6^.

### Intranasal delivery of oxytocin

Oxytocin and the oxytocin receptor antagonist L-368,899 (reported to cross the blood-brain barrier^21^) were purchased from Sigma-Aldrich (St. Louis, MO). Oxytocin (1 mg/mL) and L-368,899 (5 mg/ml) were dissolved in sterile isotonic saline. Intranasal delivery of oxytocin was performed as described previously^31^, which has recently been demonstrated to increase the oxytocin level in the brain^16^. Specifically, under isoflurane anesthesia, a 24-gauge intravenous catheter (Angiocath Plus^TM^, BD Biosciences, San Jose, CA) was inserted into the animal’s nasal cavity. To attain maximal oxytocin absorption from the nasal cavity and uptake into the cerebrospinal fluid, the rat’s head was placed at supine −70° angle position. Rats were intranasally administered either oxytocin (200 μl) or vehicle (200 μl) once per day for 2–3 days. Following the last administration, rats returned to their home cages for 2 h, after which they were subjected to the above stress treatment. In oxytocin blockade experiments, L-368,899 (5 mg/kg) or vehicle was intraperitoneally administered 1 h before the delivery of oxytocin.

### Corticosterone immunoassay

Blood samples were collected from the rat tail before stress. Immediately after stress, trunk blood was collected during the decapitation procedure. Blood serum was separated by centrifugation (5000 rpm, 20 min) and stored at −70 °C. Serum corticosterone concentrations were measured with corticosterone EIA kit (Assay Designs, Ann Arbor, Michigan).

### Hippocampal slice recordings

Two hours after the last oxytocin administration, rats (initially weighing 150–250 g) underwent one hour of stress as described above. Upon the termination of stress, rats were decapitated, their brains were rapidly extracted, and 400-μm transverse hippocampal slices were prepared. After 60–90 min of stabilization in a recovery chamber, the slices were transferred to a submersion-type recording chamber continually perfused with oxygenated artificial cerebrospinal fluid (ACSF) solution (30–32 °C) comprised of (in mM): 117 NaCl, 4.7 KCl, 2.5 CaCl_2_, 1.2 MgCl_2_, 25 NaHCO_3_, 1.2 NaH_2_PO_4_, and 11 glucose. Extracellular recordings were performed with an A-M systems Model 1800 amplifier (A-M Systems, Carlsborg, WA). A bipolar stimulating electrode (FHC, Bowdoinham, ME, USA) was placed in the hippocampal CA3 cell field to stimulate the Schaffer collateral/commissural afferents at 0.06 Hz. A glass pipette filled with ACSF (2–3 MΩ resistance) was placed in the stratum radiatum of the hippocampal CA1 field to record field excitatory postsynaptic potentials (f-EPSPs), which were digitized and analyzed using IGOR (Wave Metrics Inc., Lake Oswego, OR). Baseline responses were collected with a stimulation intensity that yielded a half-maximal response. Long-lasting LTP was induced by theta-burst stimulation (TBS): 10 stimulus trains (4 pulses at 100 Hz) delivered at 5 Hz. LTD was induced with 900 pulses delivered at 1 Hz (low frequency stimulation, LFS)^20^,^42^. The initial (negative) slope of f-EPSPs was used in statistical analyses. The magnitude of LTP was measured between 120–180 min after TBS, while the magnitude of LTD was measured 40–60 min after LFS. Statistical comparisons were conducted for group differences in averaged values of LTP or LTD magnitudes.

### Hippocampus-dependent hidden platform version of the water maze task

Vehicle or oxytocin (200 μl) was administered intranasally to male Sprague-Dawley rats (weighing 350–400 g) daily for 3 consecutive days. After the last oxytocin treatment, rats received the above stress treatment and were returned then to their home cage for about 60 min before water maze training commenced. The procedures for the hidden platform task were adapted from previous stress-water maze studies^5^,^43^. All rats received 16 massed training trials, with 15-min inter-trial interval, to find a fixed submerged platform (12 cm diameter), and escape from a circular water maze (1.83 m diameter, 58 cm high) filled with water (26 °C) made opaque with white paint (Blick Premium Tempera, Galesburg, IL). The starting points of each trial were randomly distributed across the four quadrants of the pool, and the rats were placed into the water facing the wall. If escape did not occur within 60 sec, the animal was gently guided toward the platform. On climbing onto the platform, the rats remained on the platform for 60 s, and was then moved to a holding cage for the 15 min inter-trial interval. Performance accuracy was assessed with cumulative search error on training trials. This measure was based on the distance of the animals from the escape platform throughout their search. Details of the computation were described previously^44^. Briefly, the distance of the rat from the platform was sampled 10 times per second during each trial, and then averaged in 1 -s bins. The cumulative search error is the total of the 1 s average of this proximity measure. The error is corrected for the particular start location and platform location by subtracting the proximity score that would be produced by perfect performance on that trial. The next day, a retention test (a 60 s probe trial) was given in which the platform was removed from the pool. The percentage of time spent in the annulus (3X the platform size) that contained the escape platform during training was used to assess the strength of spatial memory. A computerized tracking system (HVS Image, Hampton, UK) was used to automatically monitor the animal’s swim speed and pattern.

### Western blot analysis

Vehicle or oxytocin (200 μl) were administered to male Sprague-Dawley rats (weighing 350–400 g), which then underwent the stress protocol after the 2 h waiting period, all as described above. Afterwards, rats were rapidly decapitated and the brains removed. Dissected hippocampi were frozen at −80 °C and the proteins for the ERK and phosphorylated ERK (pERK) measurements were extracted using the standard procedure^18^. In brief, individual tissue samples were weighed and then homogenized in 500 μl of ice-cold protein lysis buffer containing 20 mM Tris–HCl (pH 7.4), 1% Triton-X 100, 1.5 mM EDTA, 40 mM KCl, 5% glycerol, 0.5 mM dithiothreitol, 1 mM NaF, 1 mM Na_3_VO_4_, 1 mM PMSF, and a proteinase inhibitor. Lysates were homogenized and then centrifuged at 18,000X g for 60 min at 4 °C. The supernatant was drawn from each sample, and an aliquot taken to determine the total protein concentration using the Bradford reagent. The proteins were then added to SDS loading buffers containing 0.1% bromophenol blue and boiled for 5 min. Each sample was then separated by SDS-polyacrylamide gel electrophoresis and transferred to a polyvinylidene fluoride (PVDF) membrane. The membrane was blocked with skim milk for 1 h and incubated with a primary antibody (Ab) against ERK (1:5000, Cell Signaling, Boston, MA) or pERK (1:2000, Cell Signaling, Boston, MA) overnight at 4 °C.It was then incubated with an anti-actin Ab (1:5000, Sigma) at room temperature. The membrane was incubated with an HRP-conjugated secondary Ab (Cell Signaling, Boston, MA) for 1 h at room temperature. The membranes were visualized using an enhanced chemiluminescence system and then developed on Hyperfilm (Amersham). The relative expression levels of all proteins were determined using a densitometer and normalized to actin expression.

### Statistical analysis

All data are expressed as mean ± standard error of the mean (SEM) and were analyzed using analysis of variance (ANOVA) applicable to the number of independent factors, and unpaired t-tests. *Post-hoc* analyses were conducted using Fisher’s least significant difference test.

## Additional Information

**How to cite this article**: Lee, S.-Y. *et al.* Oxytocin Protects Hippocampal Memory and Plasticity from Uncontrollable Stress. *Sci. Rep.* **5**, 18540; doi: 10.1038/srep18540 (2015).

## Figures and Tables

**Table 1:** Plasma corticosterone levels (ng/mL).

	Before stress	After stress
Vehicle (n = 5)	40.56 ± 18.51	296.79 ± 33.02
Oxytocin (n = 6)	53.93 ± 20.76	340.60 ± 19.7 1
